# Descending Necrotizing Mediastinitis Initially Misinterpreted as a Musculoskeletal Disorder: A Case Report

**DOI:** 10.1155/crdi/5848006

**Published:** 2026-11-05

**Authors:** Yudai Kaneda, Kenji Gonda, Akihiko Ozaki, Toyoaki Sawano, Tomohiro Kurokawa, Mitsuro Fukuhara, Hiroaki Shimmura

**Affiliations:** ^1^ Clinical Training Center Jyoban Hospital of Tokiwa Foundation Iwaki Fukushima Japan; ^2^ Department of Breast and Thyroid Surgery Jyoban Hospital of Tokiwa Foundation Iwaki Fukushima Japan; ^3^ Department of Surgery Jyoban Hospital of Tokiwa Foundation Iwaki Fukushima Japan; ^4^ Department of Chest Surgery Iwaki City Medical Center Iwaki Fukushima Japan; ^5^ Department of Urology Jyoban Hospital of Tokiwa Foundation Iwaki Fukushima Japan dlut.edu.cn

**Keywords:** case report, descending necrotizing mediastinitis, diagnostic delay, shoulder-dominant pain, *Staphylococcus aureus* bacteremia

## Abstract

Descending necrotizing mediastinitis (DNM) is a rare but life‐threatening infection in which early diagnosis is critical, although atypical symptoms and subtle initial imaging findings may delay recognition. We report a 57‐year‐old man with diabetes mellitus who presented with fever and severe right posterior neck pain radiating to the shoulder, initially leading to orthopedic evaluation; the pain was disproportionately severe relative to physical findings and refractory to analgesics, with retrosternal tenderness. Laboratory tests showed elevated C‐reactive protein with mild leukocytosis, thrombocytopenia, hypoalbuminemia, and coagulopathy, while initial noncontrast CT showed subtle findings, including pleural effusion and increased retrosternal soft tissue density. Contrast‐enhanced CT the following day revealed a mediastinal abscess, and blood cultures yielded methicillin‐susceptible *Staphylococcus aureus*; the patient improved with antimicrobial therapy without surgical drainage. This case highlights that unexplained severe pain disproportionate to physical findings should prompt consideration of deep‐seated infection and timely contrast‐enhanced imaging when DNM is suspected.

## 1. Introduction

Descending necrotizing mediastinitis (DNM) is a rare cause of acute mediastinitis, but it is among the most severe forms, with reported mortality rates of approximately 25%–40% [1, 2]. It is an inflammatory process in which infection, most commonly arising from an oropharyngeal abscess, odontogenic infection, or cervical trauma, spreads downward along the cervical fascial planes into the mediastinum [1] and typically occurs in middle‐aged to older males with underlying conditions such as diabetes mellitus or immunocompromised states [3, 4]. Despite advances in imaging, critical care, and surgical management, recent studies have reported improved mortality rates of approximately 10%–20%; however, delayed diagnosis or insufficient drainage continues to be associated with poor outcomes [5, 6].

Classically, DNM presents with fever, neck swelling, odynophagia, dyspnea, and marked inflammatory findings [7–10]. The diagnosis of DNM is commonly discussed on the basis of the criteria proposed by Estrera et al., which include clinical evidence of severe cervical infection, characteristic radiologic findings of mediastinitis, operative or postmortem confirmation of necrotizing mediastinal infection, and a demonstrable relationship between the cervical infection and mediastinitis [5].

However, early diagnosis remains challenging because initial clinical and radiologic findings may be subtle, and recognition often depends on maintaining a high index of suspicion and careful clinical assessment. Here, we report a case of clinically suspected DNM with an atypical presentation, highlighting the importance of careful physical examination and clinically guided interpretation of imaging findings for early diagnosis.

## 2. Case Presentation

A 57‐year‐old man presented to the emergency department with generalized malaise, fever, severe right posterior neck pain radiating to the right shoulder, and progressive difficulty with movement. He had a history of diabetes mellitus and was taking an SGLT2 inhibitor, a proton pump inhibitor, betahistine, and fluconazole. He lived alone and was receiving public assistance. He had no history of alcohol consumption.

Approximately 2 weeks before admission, he developed right posterior neck pain, which gradually shifted toward the right shoulder and worsened. The day before presentation, he had been evaluated by an orthopedic service and received bilateral shoulder injections, after which his symptoms reportedly worsened further.

By the day of admission, he had developed fever, profound malaise, impaired ambulation, voiding difficulty severe enough to require urinating into a plastic bottle, and fecal incontinence. In addition to severe right shoulder pain, there was erythema and tenderness over the left dorsal foot and erythema with mild swelling over the left dorsal hand.

Notably, the patient complained of extremely severe pain disproportionate to physical findings, and the pain was poorly responsive to analgesics. Marked tenderness was elicited over the anterior chest, particularly in the retrosternal area, despite the absence of clear localizing musculoskeletal abnormalities.

Admission laboratory testing showed marked inflammation, with a C‐reactive protein level of 14.47 mg/dL despite only mild leukocytosis (9300/μL), together with thrombocytopenia (6.9 × 10^4^/μL), hypoalbuminemia (2.4 g/dL), hyperbilirubinemia (total bilirubin, 3.1 mg/dL), and hyponatremia (130 mmol/L). Urinalysis showed pyuria, hematuria, bacteriuria, and glucosuria. Complete admission laboratory findings are summarized in Table 1.

**TABLE 1:** Laboratory findings on admission.

Test	Result	Reference range
*Biochemistry*		
Albumin	2.4 g/dL	4.1–5.1
Total bilirubin	3.1 mg/dL	0.4–1.5
Direct bilirubin	1.2 mg/dL	0.0–0.4
AST	25 U/L	13–30
ALT	18 U/L	10–42
ALP	73 U/L	38–113
LDH	196 U/L	124–222
γ‐GTP	46 U/L	13–64
CK	88 U/L	59–248
Blood urea nitrogen	19.3 mg/dL	8.0–20.0
Creatinine	0.86 mg/dL	0.65–1.07
Sodium	130 mmol/L	138–145
Potassium	3.8 mmol/L	3.6–4.8
eGFR	71.7 mL/min/1.73 m^2^	≥ 60

*Inflammatory marker*		
C‐reactive protein	14.47 mg/dL	< 0.14

*Complete blood count*		
White blood cell count	9300/μL	3300–8600
Red blood cell count	364 × 10^4^/μL	435–555
Hemoglobin	12.6 g/dL	13.7–16.8
Hematocrit	35.2%	40.7–50.1
MCV	97 fL	83.6–98.2
MCH	34.6 pg	27.5–33.2
MCHC	35.8 g/dL	31.7–35.3
Platelet count	6.9 × 10^4^/μL	15.8–34.8

*Urinalysis*		
Urine sediment	Negative	Negative
Urine white blood cells	30–49/HPF	< 5/HPF
Urine red blood cells	10–19/HPF	< 5/HPF
Squamous epithelial cells	< 1/HPF	< 5/HPF
Urinary tract epithelial cells	< 1/HPF	< 1/HPF
Bacteria	Positive	Negative
Urine protein	Trace	Negative
Urine glucose	4+	Negative
Urobilinogen	4+	Normal
Ketone bodies	Negative	Negative
Occult blood	3+	Negative
Urine pH	6.0	5.0–7.5
Urine color	Yellow	Yellow
Urine turbidity	Negative	Negative

Noncontrast CT of the chest and abdomen on the day of presentation did not reveal a definitive infectious focus; however, subtle abnormalities were present, including bilateral pleural effusions and mildly increased soft tissue density posterior to the sternum (Figure 1). The patient was initially managed as having an infection of unclear origin with intravenous fluids and ceftriaxone.

**FIGURE 1:**
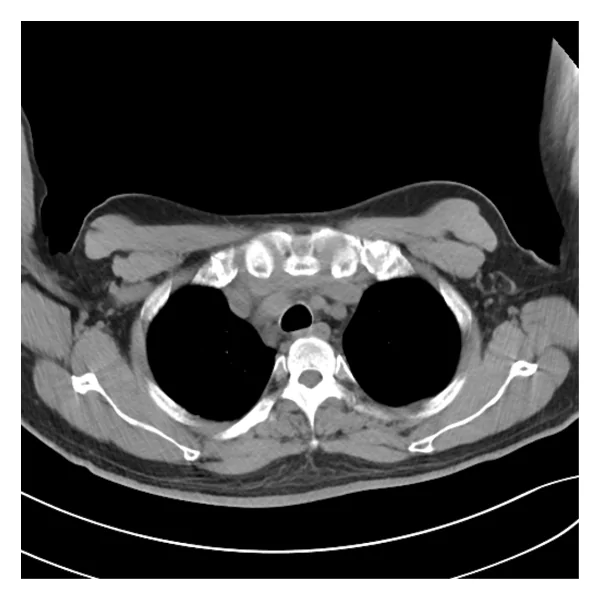
Initial noncontrast computed tomography on admission. Axial noncontrast CT demonstrates bilateral pleural effusions and mildly increased retrosternal soft‐tissue density. These abnormalities were subtle and nonspecific, and no well‐defined mediastinal abscess cavity was apparent on the initial examination.

The following day, fever persisted, and right shoulder pain worsened dramatically. At the initial evaluation, the shoulder‐dominant pain had raised consideration of a musculoskeletal disorder, including periarticular or joint infection, while pyuria and bacteriuria had raised the possibility of a urinary source of fever. However, the combination of severe pain disproportionate to the local findings, marked retrosternal tenderness, and systemic inflammation suggested a deeper infectious focus and prompted further evaluation.

On hospital day 2, repeat blood testing showed that C‐reactive protein remained high at 14.30 mg/dL, while the white blood cell count remained only 9400/μL. In contrast, the platelet count fell further to 6.0 × 10^4^/μL, sodium decreased to 128 mmol/L, albumin decreased to 2.1 g/dL, and coagulation studies revealed PT‐INR 1.48, APTT 40.5 s, and markedly elevated D‐dimer at 42.20. Contrast‐enhanced CT performed on the same day revealed bilateral pleural effusions with dependent atelectatic change and a rim‐enhancing fluid collection posterior to the sternum, suspicious for mediastinal abscess formation (Figure 2). No destructive osseous lesion was identified on the reported CT. Because of concern for a mediastinal infection requiring higher‐level source control, the patient was promptly transferred to a tertiary referral center.

**FIGURE 2:**
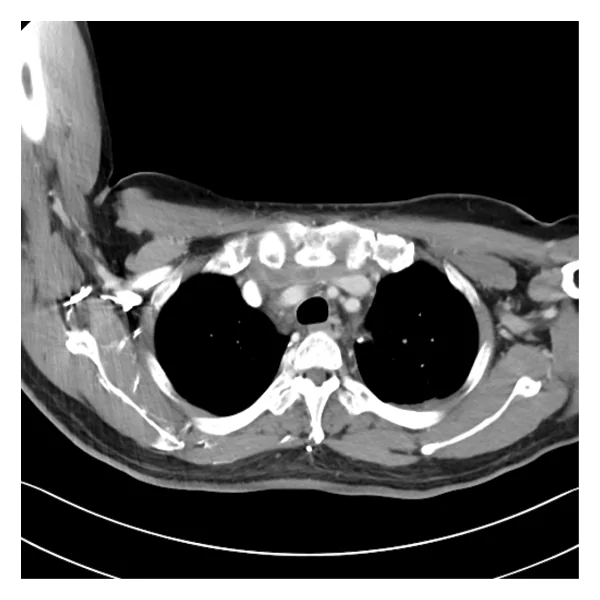
Contrast‐enhanced computed tomography obtained the following day. Axial contrast‐enhanced CT demonstrates a low‐attenuation fluid collection posterior to the sternum with peripheral rim enhancement, suspicious for mediastinal abscess formation.

Microbiological studies obtained on admission later revealed that two sets of blood cultures yielded Gram‐positive cocci identified as *Staphylococcus aureus*, with susceptibility results suggestive of methicillin susceptibility. Urine culture predominantly grew *S. aureus*, along with *Escherichia coli* and *Candida* species.

At the receiving center, the patient was initially treated with meropenem, which was subsequently de‐escalated to sulbactam/ampicillin following identification of methicillin‐susceptible *Staphylococcus aureus*. Thoracic surgical drainage, including video‐assisted thoracoscopic surgery in the event of inadequate clinical response, was considered. Clinically, retrosternal pain improved within 2–3 days after transfer, followed by gradual improvement of right shoulder pain.

At 3 weeks after transfer, inflammatory markers had gradually improved, with C‐reactive protein decreasing to 5.7 mg/dL. Follow‐up CT demonstrated that the mediastinal abscess remained localized without progression. Because both the clinical course and serial imaging showed improvement without extension of the collection, antimicrobial therapy with close clinical and radiographic monitoring was continued without surgical drainage.

Coagulation studies during hospitalization revealed decreased activity of antithrombin III, protein C, and protein S, consistent with impaired hepatic synthetic function. Underlying liver cirrhosis was suggested by hypoalbuminemia, thrombocytopenia, ascites, splenomegaly, and coagulation abnormalities. Subsequent evaluation at the referral center suggested cirrhosis secondary to nonalcoholic fatty liver disease (NAFLD) in the setting of diabetes mellitus. Portal vein thrombosis and right subclavian vein thrombosis were also identified and were considered to be associated with the underlying liver cirrhosis. Antithrombin supplementation followed by heparin anticoagulation was initiated; however, the thrombi appeared organized and did not resolve.

At the time of reporting, the patient remained under treatment primarily for liver cirrhosis, with management of ascites and edema using spironolactone, tolvaptan, and branched‐chain amino acid supplementation.

## 3. Discussion

We experienced a clinically suspected case of DNM with several atypical features, including shoulder‐dominant pain without evident cervical symptoms, subtle findings on initial noncontrast CT, and severe infection despite only mild leukocytosis.

Unlike classical DNM, which typically involves odynophagia, dyspnea, or overt neck swelling, this patient presented with progressive right posterior neck pain radiating to the shoulder, leading to initial orthopedic evaluation and bilateral shoulder injections. Importantly, the patient exhibited severe, disproportionate pain that was poorly responsive to analgesics, along with marked tenderness over the anterior chest despite the absence of clear localizing findings. Diabetes mellitus is a common comorbidity in severe deep cervical and cervicomediastinal infections, including DNM [3, 11, 12]. In the present case, the clinically striking feature was the marked discrepancy between the intensity of pain and the limited local musculoskeletal findings. This combination of otherwise unexplained severe pain, fever, and systemic inflammation should prompt consideration of a deep‐seated infectious process, including mediastinal infection.

The clinical presentation in our patient also contrasts with that in a recent Eastern European series. Vrinceanu et al. reported 26 patients with deep cervical suppurations treated at a tertiary center in Romania; 61.5% were odontogenic in origin, and common presenting manifestations included cervical swelling, neck pain, dysphagia, fever, dysphonia, dyspnea, and cervical erythema [13]. Necrotizing cervical fasciitis with DNM was present in 12 of the 26 patients, most patients required two or more cervicotomies, overall mortality was 23.1%, and diabetes mellitus was present in approximately 30% of patients [13]. In contrast, our patient had no overt cervical swelling or identified odontogenic or pharyngeal source, while severe shoulder‐dominant pain initially directed attention toward a musculoskeletal disorder. This difference further emphasizes the diagnostic challenge posed by DNM when the usual cervical manifestations are not prominent.

In addition, the infectious focus was not evident at the initial evaluation, despite subtle CT abnormalities such as pleural effusion and increased soft tissue density posterior to the sternum. Reported CT manifestations of mediastinitis include increased density and inflammatory infiltration of mediastinal fat with loss of normal fat planes, mediastinal fluid collections or gas, venous thrombosis, lymphadenopathy, and pleural or pericardial effusions [5, 14]. A well‐defined low‐attenuation collection with peripheral rim enhancement is characteristic of mediastinal abscess formation [14]. The bilateral pleural effusions and increased retrosternal soft‐tissue density seen on the initial noncontrast CT were compatible with the reported radiologic spectrum of mediastinal inflammation; however, these abnormalities were subtle and nonspecific at this stage, making mediastinal infection difficult to recognize confidently on the initial noncontrast examination. Contrast‐enhanced CT on the following day revealed a rim‐enhancing retrosternal fluid collection suspicious for an abscess. Contrast‐enhanced CT of the neck and thorax is the principal imaging modality when DNM is suspected because it better delineates inflammatory soft‐tissue changes, abscess formation, vascular complications, and the extent of mediastinal spread [5]. Therefore, when severe unexplained cervicothoracic or shoulder pain and systemic inflammation raise suspicion for deep cervical or mediastinal infection, renal function and other potential contraindications to intravenous contrast should be assessed promptly, followed by timely contrast‐enhanced CT when feasible.

The laboratory findings were discordant with infection severity: C‐reactive protein remained markedly elevated, whereas the white blood cell count showed only mild elevation, accompanied by thrombocytopenia, hypoalbuminemia, coagulopathy, and elevated D‐dimer. Cirrhosis‐associated immune dysfunction is characterized by simultaneous systemic inflammation and impairment of innate and adaptive antimicrobial immune responses; with progressive liver disease, functional immune impairment and immune paralysis become increasingly prominent [15]. This dysregulated response increases susceptibility to bacterial infection and can modify the inflammatory response to infection. In the present patient, diabetes mellitus and previously unrecognized cirrhosis attributed to NAFLD provided an important host context for the marked CRP elevation and systemic abnormalities despite only modest leukocytosis. Thus, the white blood cell count alone may underestimate the severity of infection in such patients and should be interpreted together with the clinical findings and other markers of systemic inflammation and organ dysfunction.

Blood cultures grew *S. aureus*, and *S. aureus* was also isolated from urine. Although these findings raised the possibility of hematogenous mediastinal infection, the overall clinical course was more consistent with a descending cervicomediastinal process. The patient first developed posterior neck pain approximately 2 weeks before admission, followed by progressive radiation of pain toward the shoulder and subsequently marked retrosternal tenderness, a sequence compatible with progression from the cervical region toward the mediastinum. Moreover, *S. aureus* is an uncommon cause of primary urinary tract infection, accounting for approximately 0.5%–1% of urinary tract infections, whereas *S. aureus* bacteriuria is reported in 7.8%–39% of patients with *S. aureus* bacteremia and is commonly attributed to hematogenous renal seeding [16]. Thus, the urinary isolation of MSSA was more consistent with bacteremia‐associated urinary seeding than with a typical ascending urinary tract infection. DNM is most often polymicrobial and related to odontogenic or oropharyngeal infection [3, 5]; however, *Staphylococcus* species accounted for 15.1% of single‐microbial infections in a nationwide Japanese series of surgically treated DNM [12], showing that staphylococcal infection can also occur in DNM. Taken together, the temporal progression from posterior neck to shoulder and retrosternal pain, the location of the mediastinal infection, and the microbiological findings favored clinically suspected DNM, although hematogenous mediastinal seeding remained an alternative possibility.

Early source control remains a cornerstone of DNM management, and most contemporary series emphasize drainage in addition to antimicrobial therapy [2, 3, 5]. In a nationwide Japanese study of 225 surgically treated patients, the extent of mediastinal spread was closely associated with the surgical approach and short‐term outcome, underscoring the importance of both anatomical extent and serial imaging in therapeutic decision‐making [12]. Although drainage remains the standard approach, selected reports have described successful less‐invasive management in clinically stable patients using antimicrobial therapy together with limited or image‐guided drainage [17]. In our patient, thoracic surgical intervention was considered; however, because clinical improvement was achieved with antimicrobial therapy, inflammatory markers declined, and follow‐up CT showed no enlargement of the abscess, conservative management was continued with close clinical and radiographic surveillance.

## 4. Conclusion

We report a clinically suspected case of DNM presenting with shoulder‐dominant, disproportionately severe pain and subtle findings on initial noncontrast CT. In patients with otherwise unexplained severe cervicothoracic or shoulder pain accompanied by systemic inflammation, deep cervical or mediastinal infection should be considered even when localizing findings are limited. Timely contrast‐enhanced cervicothoracic CT is important for early characterization of the infectious focus and its extent.

## Author Contributions

Conception and designing of the study: Akihiko Ozaki and Toyoaki Sawano.

Writing the paper: Yudai Kaneda.

Critical revision of the paper: Kenji Gonda, Akihiko Ozaki, Toyoaki Sawano, Tomohiro Kurokawa, Mitsuro Fukuhara, and Hiroaki Shimmura.

## Funding

The authors have nothing to report.

## Disclosure

All the authors read the final draft and approved submission.

## Ethics Statement

Institutional review board approval was not required for this single‐patient case report under the institutional policy applicable to case reports. Written informed consent was obtained from the patient for publication of this manuscript and the accompanying images, and the consent form complied with the journal requirements outlined in the author guidelines.

## Conflicts of Interest

Dr. Akihiko Ozaki received personal fees from MNES, Kyowa Kirin Inc., Becton, Dickinson and Company, Pfizer, Daiichi Sankyo Inc., and Taiho Pharmaceutical Co. Ltd., outside the scope of the submitted work. The other authors declare no conflicts of interest.

## Supporting Information

Additional supporting information can be found online in the Supporting Information section.

## Supporting information


**Supporting Information** CARE Checklist for the DNM case report (final).

## Data Availability

The data that support the findings of this study are available on request from the corresponding author. The data are not publicly available due to privacy and ethical restrictions.
