# Supplementary material for: Descending Necrotizing Mediastinitis Initially Misinterpreted as a Musculoskeletal Disorder: A Case Report
Source: Case Rep Infect Dis. 2026 Nov 5;2026:5848006. doi: 10.1155/crdi/5848006 (PMC13740666; doi:10.1155/crdi/5848006)
Supplement: Supplementary file 1 [file CRDI-2026-5848006-s001.docx]

**CARE Checklist – 2013**

**Manuscript: Descending Necrotizing Mediastinitis Initially Misinterpreted as a Musculoskeletal Disorder: A Case Report**

| **Topic** | **Item** | **CARE checklist item** | **Reported on page/line** |
| --- | --- | --- | --- |
| Title | 1 | The words “case report” (or “case study”) should be in the title along with the phenomenon of greatest interest. | p. 1, lines 1–2 |
| Keywords | 2 | Provide 2 to 5 keywords that identify areas covered in this case report, including “case report” as one of the keywords. | p. 3, lines 55–57 |
| Abstract | 3a | Introduction—What is unique about this case and what does it add to the scientific literature? | p. 4, lines 60–62 and 71–74 |
| Abstract | 3b | Main symptoms and/or important clinical findings. | p. 4, lines 62–65 |
| Abstract | 3c | The main diagnoses, therapeutic interventions, and outcomes. | p. 4, lines 66–71 |
| Abstract | 3d | Conclusion—What are the main “take-away” lessons from this case? | p. 4, lines 71–74 |
| Introduction | 4 | Summarize why this case is unique with references to the relevant medical literature. | pp. 5–6, lines 78–100 |
| Patient information | 5a | De-identified demographic and other patient-specific information. | p. 6, lines 103–107 |
| Patient information | 5b | Primary concerns and symptoms of the patient. | pp. 6–7, lines 103–117 |
| Patient information | 5c | Medical, family, and psychosocial history including relevant genetic information. | p. 6, lines 105–107 (medical and psychosocial history; family/genetic history not specifically reported) |
| Patient information | 5d | Relevant past interventions and their outcomes. | p. 6, lines 105–112 (medications and pre-presentation shoulder injections) |
| Clinical findings | 6 | Describe significant physical examination and important clinical findings. | p. 7, lines 115–122 |
| Timeline | 7 | Depict important information from the patient’s history as a timeline (figure or table). | pp. 6–10, lines 109–190. The clinical course is presented chronologically in the Case Presentation rather than as a separate timeline table. |
| Diagnostic assessment | 8a | Diagnostic methods (e.g., physical examination, laboratory testing, imaging, surveys). | pp. 7–9, lines 119–161 |
| Diagnostic assessment | 8b | Diagnostic challenges (e.g., access, financial, or cultural). | pp. 7–9, lines 119–155; pp. 11–13, lines 193–241 (atypical shoulder-dominant pain and subtle initial CT findings) |
| Diagnostic assessment | 8c | Diagnosis, including other diagnoses considered. | p. 8, lines 137–143; pp. 8–9, lines 145–155 |
| Diagnostic assessment | 8d | Prognostic characteristics (e.g., staging) where applicable. | No formal staging/prognostic score was used; relevant host factors are described on p. 10, lines 177–186 and pp. 13–14, lines 243–256. |
| Therapeutic intervention | 9a | Types of therapeutic intervention (e.g., pharmacologic, surgical, preventive, self-care). | pp. 8–10, lines 134–175 and 184–190 |
| Therapeutic intervention | 9b | Administration of therapeutic intervention (e.g., dosage, strength, duration). | pp. 8–10, lines 134–175. Antimicrobial agents, route where stated, and treatment sequence are reported; exact doses/durations are not specified. |
| Therapeutic intervention | 9c | Changes in therapeutic intervention, with rationale. | pp. 9–10, lines 163–175 (de-escalation after MSSA identification; drainage considered but deferred with clinical/radiographic improvement) |
| Follow-up and outcomes | 10a | Clinician- and patient-assessed outcomes (when appropriate). | pp. 9–10, lines 167–175 |
| Follow-up and outcomes | 10b | Important follow-up diagnostic and other test results. | p. 10, lines 170–175 |
| Follow-up and outcomes | 10c | Intervention adherence and tolerability (and how this was assessed). | Not specifically reported. |
| Follow-up and outcomes | 10d | Adverse and unanticipated events. | Not specifically reported. |
| Discussion | 11a | A scientific discussion of the strengths and limitations associated with this case report. | pp. 11–16, lines 193–290. As this is a single-patient case report, the discussion focuses on case-specific diagnostic and therapeutic insights; etiologic uncertainty and management context are addressed particularly at lines 258–290. |
| Discussion | 11b | Discussion of the relevant medical literature. | pp. 11–16, lines 197–290 |
| Discussion | 11c | The rationale for conclusions (including assessment of possible cause and effect). | pp. 14–16, lines 258–290 |
| Discussion | 11d | The primary “take-away” lessons of this case report. | p. 16, lines 293–298 |
| Patient perspective | 12 | The patient should share their perspective or experience whenever possible. | Not reported. |
| Informed consent | 13 | Did the patient give informed consent? Please provide if requested. | pp. 2–3, lines 36–41 |

*Note: This checklist reflects the content actually reported in the revised manuscript. Items not specifically reported are identified as such rather than inferred.*
